# Modeling Sepsis: Establishment and Validation of a 72-Hour Swine Model of Penetrating Abdominal Trauma

**DOI:** 10.3390/medicina61091523

**Published:** 2025-08-25

**Authors:** Catharina Gaeth, Travis R. Madaris, Jamila Duarte, Alvaro Rodriguez, Matthew D. Wegner, Amber Powers, Randolph Stone

**Affiliations:** 1 Combat Wound Care, United States Army Institute of Surgical Research, Fort Sam Houston, San Antonio, TX 78234, USA; 2 DoD Food Analysis and Diagnostic Laboratory, Public Health Command, West, Fort Sam Houston, San Antonio, TX 78234, USA

**Keywords:** penetrating abdominal trauma, fecal peritonitis, preclinical trauma models, sepsis

## Abstract

*Background/Objectives:* Fecal peritonitis following penetrating abdominal trauma is a serious condition that often results in sepsis and organ failure. The aim of our study was to develop a novel conscious porcine model of sepsis and organ dysfunction caused by multiple penetrating injuries to the small and large intestines. *Methods:* Twelve female Yorkshire pigs (average weight 50.6 ± 6.5 kg) were divided into two groups: Penetrating Abdominal Trauma (PAT) (*n* = 8) and Control (*n* = 4). All surgical procedures were performed under anesthesia with adequate analgesia. In the PAT group, the small and large intestines were punctured, and feces mixed with saline were introduced into the abdominal cavity to induce peritonitis. The Control group received sham surgery with only saline solution. The animals were observed in a conscious state over a period of 72 h, vital parameters were recorded, and blood samples were taken regularly. We adapted a pig-specific SOFA score and developed pig-specific SIRS criteria and NEWS2 score to assess organ function. The model was validated by independent investigators. *Results:* The survival rate in the PAT group was 75%, with an average survival time of 58.5 h, while all animals in the Control group survived to euthanasia. Monitoring showed pathophysiological changes, such as tachycardia, leucopenia, and thrombocytopenia, indicative of sepsis and organ dysfunction. Blinded investigators independently confirmed the model’s validity. *Conclusions:* A new swine model of penetrating abdominal trauma and sepsis has been successfully developed that demonstrates significant physiological and immunologic changes comparable to human sepsis. This new model provides a realistic platform for future research into sepsis, its diagnostics, and the evaluation of therapeutic strategies.

## 1. Introduction

Penetrating abdominal trauma, a severe and potentially life-threatening injury pattern, is particularly prevalent in military settings, where it is predominantly caused by gunshot wounds. Such injuries often result in significant abdominal tissue damage and can lead to fatal outcomes [1,2,3]. Data from the Department of Defense Trauma Registry indicate that abdominal trauma accounted for 7–17% of all combat casualties during conflicts in Iraq and Afghanistan between 2002 to 2016 [4,5,6]. Commonly affected areas include the gastrointestinal tract, including the small and large bowel, stomach, duodenum, and rectum, often characterized by multiple, up to five, full-thickness abdominal gunshot wounds. Of critical concern in these injuries is the rapid development of peritonitis due to chemical, enzymatic, or bacterial contamination [7]. In addition, the risk of complications, particularly intra-abdominal infections and wound infections, is substantial and can progress into sepsis if untreated, significantly increasing morbidity and mortality [1,4]. Therefore early, fast, and accurate diagnosis and treatment are imperative [8]. Current military treatment strategies focus on damage control surgery, resuscitation, and antibiotic therapy to stabilize patients and prevent further contamination in conjunction with rapid evacuation to advanced surgical facilities [9,10]. However, anticipated future multi-domain operations are expected to delay or prevent such evacuation, potentially increasing mortality and complication rates related to sepsis, particularly progression to systemic inflammatory response syndrome (SIRS) [11,12]. 

SIRS refers to the body’s own system-wide internal response to infection or severe trauma, initiated by an early immunological response involving cytokines such as TNF-α and IL-6, which triggers a proinflammatory reaction [13]. This cytokine cascade activates immune cells and, when elevated to a certain level, initiates specific symptoms, such as fever, correlating with infection severity [14,15]. Detection in clinical practice is often limited due to varying serum levels of individual cytokines during the initial infection phase. However, specific SIRS criteria help to assess the patient’s condition and recognize pathologic changes at an early stage [16,17]. Additionally, the SOFA (Sequential Organ Failure Assessment) score is considered the gold standard for evaluating disease severity and mortality risk in cases of sepsis [18,19]. It is calculated from six physiological parameters that reflect key organ functions: coagulation (platelet count), renal (creatinine), hepatic (total bilirubin), circulatory (mean arterial pressure, MAP), pulmonary (PaO_2_/FiO_2_ ratio), and neurological (consciousness and responsiveness) [20]. Nonetheless, the reliability of the SOFA score is debatable, which has led to the use of alternative scoring systems, such as the National Early Warning Score 2 (NEWS2). NEWS2 is used in the evaluation of sepsis in cases where only basic diagnostic instruments are available, such as those measuring respiration rate, oxygen saturation, systolic blood pressure, pulse rate, level of consciousness, and temperature [21,22]. 

Various disease models have been utilized to address these challenges and enhance the understanding of the pathophysiology of abdominal injuries and sepsis. Particularly, porcine models have been used due to their anatomical and physiological similarity to humans [23,24]. However, existing models present certain limitations. For instance, sepsis is induced heterogeneously through methods such as cecal ligation and/or puncture or the administration of live bacteria or exogenous toxins [25]. Additionally, these models often focus on hemorrhage, resuscitation, and peritonitis. Furthermore, these published animal models often fail to translate to humans because they do not adhere to the established clinical standards and criteria for diagnosing sepsis, such as species-specific SIRS criteria and SOFA score [26]. Notably, published sepsis models typically only investigate the acute phase of sepsis, which occurs within the first 12 to 24 h after sepsis is induced [27,28,29]. These models fail to consider delayed patient care and long-term outcomes, up to 72 h. Moreover, during described short-term investigation, the animals are kept under continuous anesthesia. Therefore, the impact of anesthesia must be considered when interpreting the results of published studies, as anesthetics influence bodily physiology. For instance, blood pressure and heart rate are artificially reduced, respiration rate is controlled, and locomotion and neurological function assessments are limited [30,31]. These factors together limit the monitoring of critical early indicators of sepsis and the applicability of the results to real-life settings, underscoring the need for a large animal model of fecal peritonitis that considers a prolonged investigation period in animals that are not under the influence of anesthetics.

Therefore, the aim of this study is to establish and validate a conscious sepsis porcine model of penetrating abdominal injury with fecal peritonitis that simulates delayed patient treatment and examines pathophysiology for up to 72 h post-injury. The study only uses anesthesia for the surgical procedures. It provides a relevant preclinical framework for optimizing the care of patients. To improve the translation to humans, the clinically relevant diagnostic scoring systems (SIRS, SOFA, and NEWS2) are adapted to species-specific parameters. 

## 2. Materials and Methods

### 2.1. Animals

Research was conducted in compliance with the Animal Welfare Act, implementing animal welfare regulations and the principles of the Guide for the Care and Use of Laboratory Animals. The Institutional Animal Care and Use Committee approved all research conducted in this study (protocol #A-22–009 approved 29 April 2022). The facility where this research was conducted is fully accredited by AAALAC International. A model development phase utilized 6 animals to optimize the number and location of injuries as well as post-operative care for a successful model. For the validation study, 12 female Yorkshire pigs (Midwest Research Swine, Gibbon, MN, USA), aged 6–9 months with an average weight of 50.6 ± 6.5 kg were used. Animals were included in all data analysis as long as they survived and recovered from injury. One animal was excluded that did not recover from surgical instrumentation. To reduce animal stress, the animals were acclimated to the facility for a minimum of seven days and received behavioral training with human handling for a minimum of two weeks prior to surgery [32]. The experimental details are reported following the ARRIVE (Animal Research: Reporting of In Vivo Experiments) guidelines. 

### 2.2. Anesthesia and Analgesics

Animals had ad libitum access to food and water, except during fasting for anesthesia. Anesthesia was induced according to standardized protocols at the Institute [23,24]. Briefly, glycopyrrolate (0.01 mg/kg) was injected intramuscularly (IM) into the neck as needed to reduce salivation and vagal bradycardia. Tiletamine-zolazepam (4–6 mg/kg, IM) or ketamine (10–25 mg/kg, IM) was used for the induction of anesthesia. Anesthesia began with 3–5% (*v*/*v*) isoflurane in oxygen via a face mask. Animals were intubated and ventilated with a tidal volume of 8–12 mL/kg, a peak pressure of 20 cm H_2_O, and 8–20 breaths per minute (bpm), with end-tidal PCO_2_ maintained at 40 ± 5 mmHg. Anesthesia was maintained with 1–3% isoflurane in oxygen. Buprenorphine sustained release (Bup SR, 0.1–0.24 mg/kg) was administered to all animals subcutaneously into the dorsal lumbar spine, lateral neck, or caudal thigh muscles before all surgeries. Additional analgesia for breakthrough pain after surgery was administered depending on daily pain assessments.

### 2.3. Surgical Instrumentation

Three days prior to experimental injury, in anesthetized animals with adequate analgesia, catheters to be used later for scheduled blood draws were placed in both the carotid artery and jugular vein and tunneled to exit out the back of the neck. A DSI (Data Sciences International, New Brighton, MN, USA) telemetry implant was placed into the femoral artery to record activity, blood pressure, temperature, and derived parameters heart rate (HR) and respiration rate. The DSI transmitter was sutured subcutaneously under the adjacent skin. Wounds were covered in sterile gauze and secured in place with Tegaderm. A pig jacket (Lomir) with a pocket (to protect the catheters) on the dorsum was applied to each animal and they were allowed to recover in their pen. 

### 2.4. Abdominal Injury and Fecal Peritonitis

Animals were randomized into a Control (*n* = 4) or Penetrating Abdominal Trauma (PAT, *n* = 8) group. On the day of injury, in anesthetized animals with adequate analgesia, a sterile abdominal incision (laparotomy) was made along the midline, approximately 2 cm cranial to the umbilicus. In the PAT group, 10 mm biopsy punches were made in both the small and large intestines (five punches in each) to mimic gunshot wounds. Any bleeding was mitigated by compression to avoid hemorrhage-associated pathologies. Autologous feces (1 g/kg body weight) were expressed from the puncture sites, diluted with 100 mL of 37 °C saline, and administered into the abdominal cavity through a drain tube. The puncture sites were left open to mimic persistent fecal leakage from the intestines. Control animals had sham surgery and only received 100 mL of 37 °C saline to the abdominal cavity with no organ puncture. In both groups, the abdominal incisions were closed with sutures; wound dressings and pig jackets were reapplied. Animals recovered from anesthesia and were transferred to their pen. Blood samples were collected from conscious animals pre- and post-injury and every eight hours afterwards. Computed tomography (CT) imaging was performed on anesthetized animals before injury and immediately prior to euthanasia. Seventy-two hours after injury, the surviving animals underwent anesthesia and euthanasia with an overdose of pentobarbital (Fatal Plus, at least 150 mg/kg intravenously). 

### 2.5. Establishment of SIRS Criteria for Yorkshire Pigs

The SIRS criteria for pigs were determined using values from 48 healthy animals across three studies with identical timelines. All animals underwent the same surgical instrumentation as stated above at least three days prior to injury. The HR, temperature, and respiration rate were collected from the DSI telemetry device and analyzed using Ponemah V6.51 (Supplementary Material S1), representing normal values in healthy animals. Accounting for the 12-h light cycle, all data from the 48-h period immediately prior to the baseline blood draw on the morning of injury were included. The white blood cell (WBC) values were obtained from the blood draws that occurred during the same 48-h period to include the baseline blood draw the morning before injury. We considered the data collected during this period to best represent normal values in healthy animals of this strain, sex, and weight. The criteria for a SIRS state were determined using these normal average values ± 2 standard deviations and considered diurnal oscillations in HR, temperature, and respiration rate (Table 1) [33]. 

### 2.6. Sequential Organ Failure Assessment (SOFA) Score for Yorkshire Pig

As the development and progression of sepsis are some of the fatal complications of penetrating abdominal trauma, the results were analyzed based on adapted criteria from previous publications by Waterhouse et al. and Fukuda et al. [34,35]. Fukuda et al. developed a neurological evaluation for sheep, which was altered for swine after consultation with our veterinarian and termed the swine neurological observed response test (SNORT) [35]. For details on the SOFA and SNORT assessments, see Table 2 and Supplementary Material S2.

### 2.7. National Early Warning Score 2 (NEWS2) for Yorkshire Pigs

The National Early Warning Score 2 from the National Health System UK was used to evaluate deterioration and the septic state of Yorkshire pigs (Table 3) [36]. Values were generated as described in Section 2.5, using normal average values ± 2 standard deviations under consideration of diurnal oscillations.

### 2.8. Blood Work, Pathology, and Imaging

Blood chemistry and complete blood counts were performed by the Laboratory Support Group at the Institute according to standardized protocols on file and as previously described [37]. An iSTAT^®^ analyzer with CG4+ cartridges (Abbott) was used to measure pH and PaO_2_, as previously described [37]. For immunoprotein analysis, a Luminex Platform (Bio-Rad, Waltham, MA, USA) was used per the manufacturer’s guidelines. Blinded histological evaluation of organ samples and imaging was conducted by a board-certified pathologist at the Dept. of Defense Food Analysis and Diagnostic Laboratory (Public Health Command, West). Computed tomography was conducted on anesthetized pigs using contrast (Isovue-370; Iopamidol 755 mg/mL; contains sodium 0.053 mg, organically bound iodine 370 mg/mL).

### 2.9. Penetrating Abdominal Trauma Model Validation

Five independent investigators (a clinician, a researcher, and two experienced and one unexperienced animal technician) reviewed vitals, blood work values, pain assessments, and neurological assessments of the 12 animals with all classification data redacted. The blinded reviewers sorted the animals into the PAT or Control group and included their justification.

### 2.10. Statistical Analysis

Power analysis and literature review guided our sample size. Statistical testing was performed using GraphPad Prism 10.4 (GraphPad Software LLC., La Jolla, CA, USA). Outliers were removed if they were identified with the GraphPad ROUT Method with Q = 1%. Mixed-effects analysis with Šídák’s multiple comparisons test was used for telemetry data and blood work data. This was necessary to account for missing values (two animals did not survive until the 72-h endpoint). Data are reported as the average ± standard deviation. For categorical datasets (SNORT, SIRS, SOFA, and NEWS2), the nonparametric multiple Mann–Whitney test was used to compare mean rank scores with the Holm–Šídák method to correct for multiple comparisons; these data are reported as the median [interquartile range (IQR)]. The *p*-value was determined for each parameter comparing the Control group to the combined injury group with an α = 0.05 with statistical significance of *p* < 0.05. Exact *p*-values for graphs can be found in Supplementary Material S3.

## 3. Results

### 3.1. General Model Characteristics and Pathology

**Figure 1:**
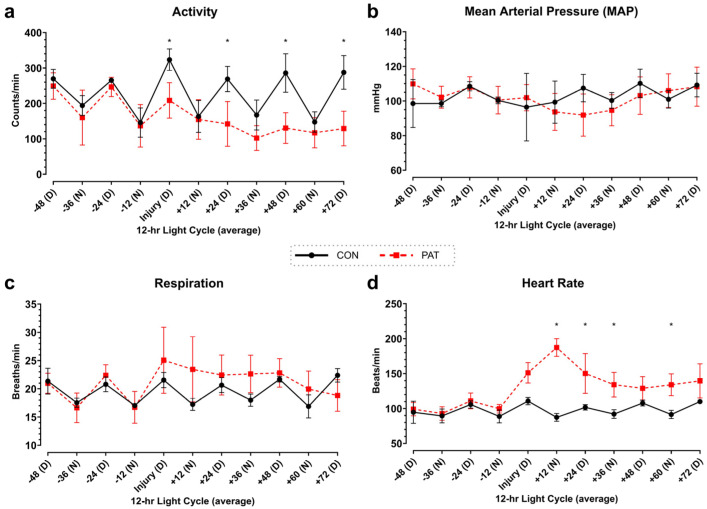
Diurnal cycling of activity and physiological vitals: telemetric analysis of (**a**) activity, (**b**) MAP, (**c**) respiration, and (**d**) HR. To account for the diurnal cycle, the values were averaged throughout the 12-h period (D = day and N = night). Control (CON) shown in black with circles and Penetrating Abdominal Trauma (PAT) shown in red with squares. Data are presented as mean ± SD, * *p* < 0.05. Control group: *n* = 4. PAT group: *n* = 8.

The survival rate in the PAT group was 75% (*n* = 6), with an average survival time of 58.5 h. All animals in the Control group survived to euthanasia. The activity and other vitals were monitored (Figure 1). Control animals exhibited diurnal fluctuations in parameters, a pattern that was absent in the injury group. Specifically, the activity was significantly reduced during the daytime cycles (Figure 1a). 

**Figure 2:**
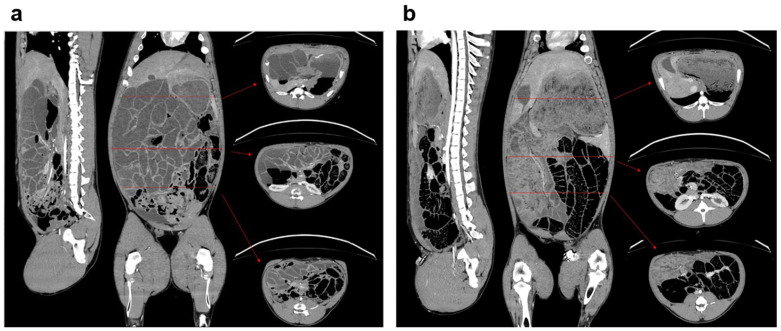
Comparison of computed tomography (CT) of abdomen after 72 h: sagittal, coronal, and axial planes of (**a**) Penetrating Abdominal Trauma (PAT) group vs. (**b**) Control group. Representation of free fluid and air in the peritoneal cavity with peritoneal thickening in animals of the PAT group. Red arrows indicate the location of the axial plane.

In addition to the macroscopically visible changes in the abdominal cavity, i.e., fecal distribution, fibrin coating of the organs, and gray-colored avital intestinal loops, CT images, taken immediately before euthanasia, showed signs of fecal peritonitis with a thickening of the peritoneum, distended intestinal loops, and free fluid as well as air in the abdominal cavity (Figure 2).

**Figure 3:**
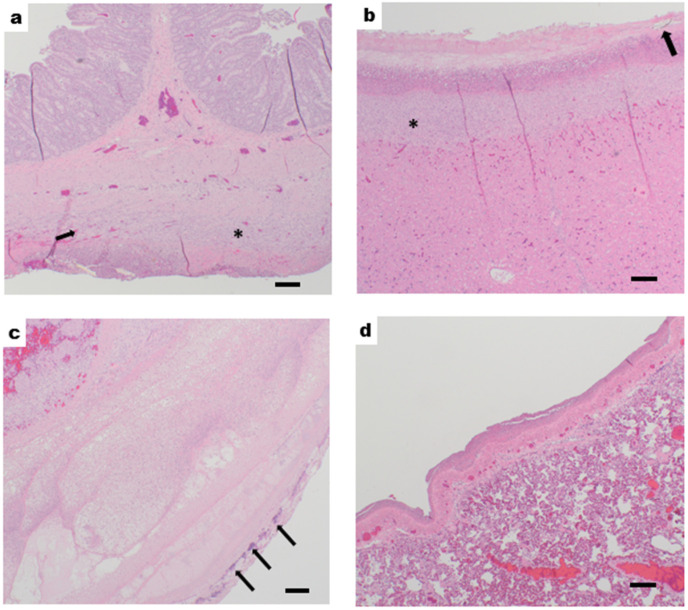
Histological examination, representative H&E staining: (**a**) jejunum, (**b**) liver, (**c**) spleen, and (**d**) lung from the PAT group taken at 40X magnification. (**a**) Jejunum: subacute, focally extensive, moderate serositis with necrosis, fibrin, neutrophils, fibrinonecrotic debris, and embedded plant material (arrow). (**b**) Liver: subacute, diffuse, marked serositis and hepatitis with necrosis, fibrin, neutrophils, fibrinonecrotic debris, and embedded plant material (arrow). (**c**) Spleen: acute, diffuse, severe serositis with necrosis, fibrin, neutrophils, fibrinonecrotic debris, severe necrosis, and edema. Colonies of bacteria are indicated (arrows). (**d**) Lung: acute, diffuse, moderate serositis with necrosis, fibrin, neutrophils, and fibrinonecrotic debris. (*) indicates early granulation tissue and mononuclear cell infiltration. Scale bar = 500 µm.

Histological examination in the PAT group revealed that 7 of 8 animals exhibited acute to subacute polyserositis with necrosis, fibrin, neutrophils, fibrin necrotic debris, and rarely early granulation tissue and mononuclear cell infiltration across organs such as the jejunum, liver, spleen, and lung (Figure 3). Most also exhibited varying degrees of neutrophilic lymphadenitis with necrosis and hemorrhage. The heart and kidney remained normal in all animals. The examined tissues of the Control group showed no significant lesions or pathological changes.

### 3.2. Diagnostic Scoring 

#### 3.2.1. SIRS Parameters

**Figure 4:**
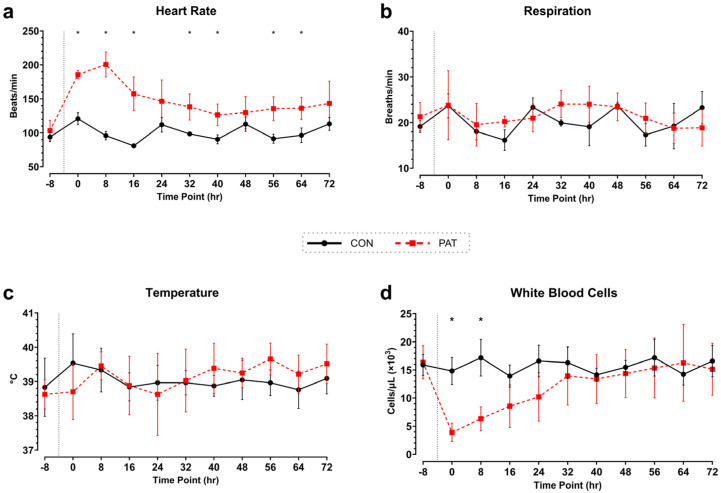
SIRS criteria in Penetrating Abdominal Trauma group vs. Control group: (**a**) heart rate (HR), (**b**) respiration, (**c**) temperature, and (**d**) white blood cell count (WBC, from whole blood). Telemetry data averages for every 8-h timepoint, corresponding with each blood draw (**a**–**c**). The average WBC count before surgery and every 8 hr timepoint post-surgery (**d**). Dotted line represents approximate time of injury. Control (CON) shown in black with circles and Penetrating Abdominal Trauma (PAT) shown in red with squares. Data are presented as mean ± SD, * *p* < 0.05. Control group: *n* = 4. PAT group: *n* = 8.

The HR in the PAT group increased significantly after injury, with the highest value reached after 8 h (201 ± 18 bpm, *p* = 0.007, Figure 4a) The average HRs after surgery were 150 ± 25 bpm in the PAT group and 101 ± 13 bpm the Control group. By contrast, the respiratory rate showed no significant differences (*p* > 0.990) between the two groups (post-injury average, 20 breaths/min ± 3 vs. 21 ± 2, PAT vs. Control, Figure 4b). Additionally, temperature also showed no significant changes (post-injury average, 39.2 °C ± 0.4 vs. 39.0 ± 0.2, PAT vs. Control, Figure 4c). The baseline count of white blood cells (WBCs) was similar (*p* > 0.999) between the PAT (16.4 × 10^3^ cells/µL ± 3.0) and Control groups (15.9 × 10^3^ cells/µL ± 1.9). Pronounced leukocytopenia was observed in the PAT group within the first 8 h post-injury (3.9 × 10^3^ cells/µL ± 1.6, *p* = 0.008, Figure 4d). Through 40 h, there was a gradual increase in WBC count that approached that of the Control group. By the end of the study, the WBC count did not differ (*p* > 0.999) between the two groups (15.1 × 10^3^ cells/µL ± 4.6 compared to 16.6 ± 2.8, PAT vs. Control, Figure 4d).

#### 3.2.2. SOFA Parameters 

**Figure 5:**
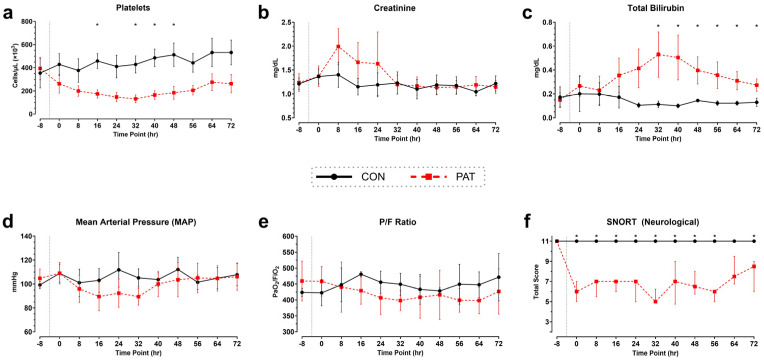
SOFA criteria in Penetrating Abdominal Trauma group vs. Control group: (**a**) Platelet count, (**b**) creatinine concentration, and (**c**) total bilirubin from whole blood. The values are the averages at each individual blood draw before and after injury. (**d**) Mean arterial pressure (MAP) recorded through telemetric implant. (**e**) P/F-ratio (ratio between arterial O_2_ partial pressure (PaO_2_) and fraction of inspired (Fi) O_2_. Data are presented as mean ± SD. (**f**) Neurological assessment, SNORT (swine neurological observed response test), of animals before and after surgery. Data are presented as median [IQR]. Dotted line represents the approximate time of injury. Control (CON) shown in black with circles and Penetrating Abdominal Trauma (PAT) shown in red with squares. * *p* < 0.05. Control group: *n* = 4. PAT group: *n* = 8.

The key physiological parameters of the SOFA score are displayed in Figure 5. Animals in the PAT group exhibited thrombocytopenia after injury, which decreased over time to a minimum platelet count of 132.2 × 10^3^ cells/µL ± 33.5 (*p* = 0.022). After 32 h, the platelet count started to increase but failed to return to baseline levels (Figure 5a). Creatinine levels increased in the PAT group in the first 24 h after injury, peaking at 2.0 ± 0.4 mg/dL (*p* = 0.146). After 32 h, creatinine levels had returned to baseline values and were similar to those of the Control group (*p* > 0.999, Figure 5b). Total bilirubin levels demonstrated a delayed increase after injury in the PAT group, peaking at 0.53 ± 0.19 mg/dL, compared 0.11 ± 0.03 mg/dL in the Control group (*p* = 0.029, Figure 5c). The MAP and P/F ratio trended lower in PAT than Control after surgery. The post-surgery average MAP was 100 ± 7 mmHg in the PAT group vs. 106 ± 4 mmHg in the Control group and the P/F-ratio was 418.15 ± 20.28 in the PAT group and 448.61 ± 18.04 in the Control group (Figure 5d,e). Animals in the PAT group had a reduced neurological responsiveness. The median SNORT score was normal (11) in the Control group but was significantly decreased (*p* < 0.05) in all timepoints after injury except at 64 h (*p* = 0.066) in the PAT group. The post-injury median score in the PAT group was 6.6 [5.9, 7.1] (Figure 5f).

#### 3.2.3. Comparison of Diagnostic Scoring 

**Figure 6:**
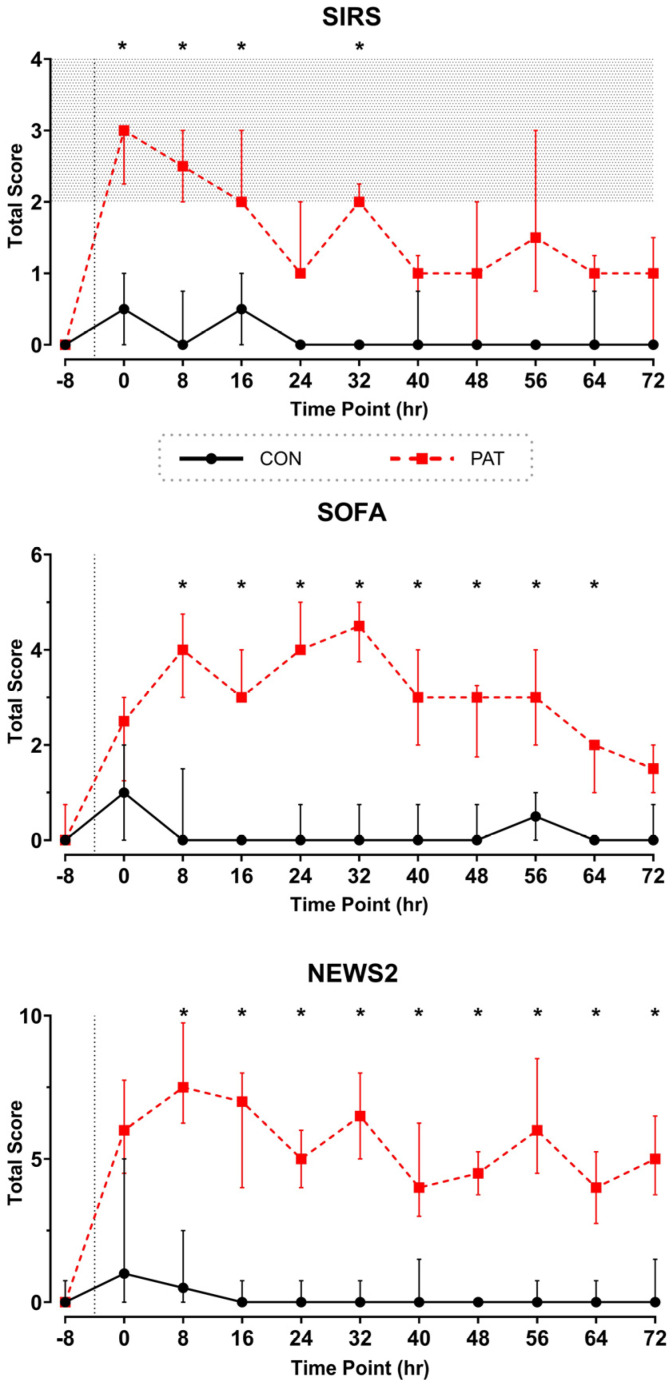
Results of diagnostic scoring: (**a**) total SIRS score, (**b**) total SOFA score, and (**c**) NEWS2 score. Derived from parameters at every 8 h. Shading indicates SIRS state (a). Control (CON) shown in black with circles and Penetrating Abdominal Trauma (PAT) shown in red with squares. Data are shown as median [IQR]. In all panels * *p* < 0.05. Control group: *n* = 4 animals. PAT group: *n* = 8.

With the development of the pig-specific diagnostic criteria, it is possible to define and classify the severity of the disease in animals. To meet the SIRS state, two of the four parameters have to meet the criteria. The PAT group met these conditions for the first 16-h after injury (Figure 6a). During this window, the median SIRS score was significantly (*p* < 0.05) different than that of the Control group and remained elevated throughout the study. Importantly, the SIRS thresholds we determined did not categorize Control animals in a SIRS state (Table 4). Using the SOFA scoring system, a similar trend to SIRS scoring was observed (Figure 6b). The median SOFA for the Control group was 0 at all timepoints except for immediately after surgery (1.0 [0, 2.0]). A key observation was an overall increase in the median SOFA score in the PAT group during the first 32 h. After 72 h, 50% of the PAT group still fulfilled the SOFA criteria compared to none of the Control group animals (*p* < 0.05, Table 4). Taking the NEWS2 score into account (Table 4), the results in the PAT group showed significantly higher values from the first 8 h to the end of the protocol than in the Control group (Figure 6c). Animals in the PAT group remained in a state of acute illness, as defined by a score of ≥5 or an extreme value variation. Except at the timepoint of 64 h, all animals in the PAT group exhibited at least one extreme value variation.

### 3.3. Blood Biochemistry Laboratory and Protein Results 

Examination of blood for electrolytes generally revealed a lower concentration of electrolytes in the PAT group after injury (Supplementary Material S4). Pronounced hyperlactatemia was observed acutely after surgery in the PAT group (6.3 ± 2.4 mM) but not in the Control group (1.4 ± 0.3, *p* = 0.009, Supplementary Material S4). Glucose levels in the PAT group initially increased significantly after injury (174 ± 36 mg/dL, *p* = 0.053); however, this was followed by a hypoglycemic state from up to 32 h after injury. In alignment with the elevated bilirubin levels (Figure 3c), AST (aspartate transaminase) levels trended higher starting at 8 h after injury. Similarly, the renal function-associated parameter, blood urea nitrogen (BUN), was also elevated after injury (Supplementary Material S4). 

**Figure 7:**
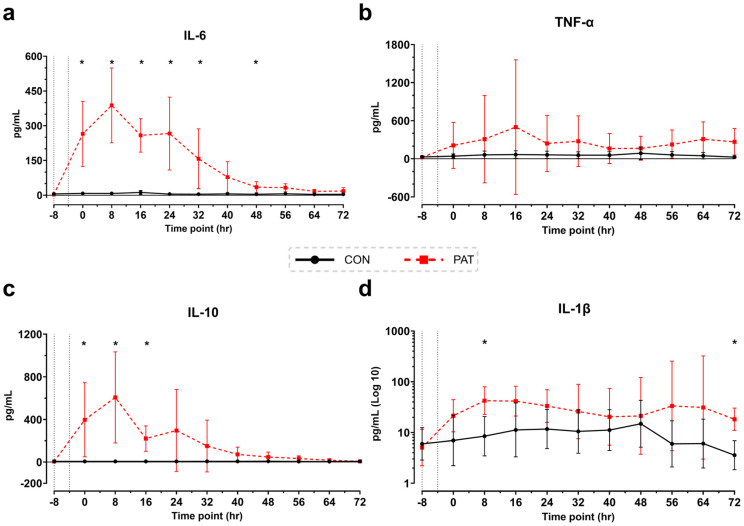
Cytokine results baseline to 72 h: (**a**) IL-6, (**b**) TNF-α, (**c**) IL-10, and (**d**) IL-1β. Dotted lines represent baseline levels at −8 and approximate time of injury. Control (CON) shown in black with circles and Penetrating Abdominal Trauma (PAT) shown in red with squares. Data are presented as mean ± SD, * *p* < 0.05. Control group: *n* = 4. PAT group: *n* = 8.

Significant differences were observed at several timepoints in the immunoproteins interleukin (IL)-6, IL-10, and IL-1β (Figure 7). The TNF-α level trended higher after injury in the PAT group compared to the Control group (Figure 7b) but was not statistically significant. TNF-α levels remained elevated throughout the study, while levels of IL-6, IL-10, and IL-1β increased significantly after injury, peaking at 8 h (Figure 7a, 7c, and 7d). IL-6 levels remained significantly elevated until 32 h. IL-6 and IL-10 levels decreased within 48 h after injury, and by 72 h they were close to normal in the PAT group. The IL-1β level remained elevated until the end of the 72-h observation period (Figure 7d).

### 3.4. Model Validation 

Four out of five blinded reviewers correctly assigned the corresponding group to all 12 animals. One reviewer misclassified an injured animal as a Control, and when informed of the mistake, the second trial was correct. Collectively, averaging all five reviewers’ success rates resulted in ~98% correct identification.

## 4. Discussion

### 4.1. Model Establishment and Validation 

This sepsis model of fecal peritonitis from penetrating abdominal trauma represents a significant advance in translational sepsis research. It realistically depicts the injury patterns and pathophysiological consequences of abdominal gunshot wounds in a setting of delayed patient care. Further, the ability of this model to mimic the clinical features of severe sepsis as observed in human patients can contribute to a deeper understanding of disease progression in a controlled experimental setting and helps to evaluate therapeutic interventions more accurately [38,39]. Key to this work is the novel adaptation of clinical sepsis scores—SIRS, SOFA, and NEWS2—for swine. The SIRS criteria for Yorkshire swine were defined for the first time, using baseline data to account for physiological differences, enabling systematic assessment of sepsis severity and organ impact in swine. This sets a precedent for future translational research, where such adaptations are crucial to ensure validity and applicability to human conditions. Furthermore, this novel model is distinguished by its extended follow-up duration, mimicking prolonged time to patient assessment and treatment, up to 72 h. This feature enables the observation and depiction of the gradual progression of fecal peritonitis, ultimately leading to the development of fulminant sepsis. Independent validation of the model by blinded investigators adds another layer of robustness and reliability to our study. The fact that blinded investigators were able to accurately assess and categorize the clinical and diagnostic parameters of the model increases the credibility of the model and underscores its potential for broad application in future research. 

### 4.2. Organ Dysfunction, Biochemical and Immune Responses

Our findings on organ dysfunction and the associated biochemical and immunological changes provide valuable insights into abdominal sepsis pathophysiology. One of the most important observations was the significant HR increase in the PAT group, peaking 8 h post-injury and staying elevated throughout the 72-h observation period (Figure 4). This sustained tachycardia mirrors cardiovascular changes in human sepsis, where increased HR is a compensatory mechanism to maintain essential body functions [38,40]. The replication of this physiological response in our porcine model emphasizes the importance of this model and its potential as a tool to study cardiovascular changes in sepsis. No significant changes were observed in MAP, respiration rate, or temperature. 

Renal impairment was suggested by an increase in creatinine and BUN levels. Changes in urine output were not measurable in this study because the animals were awake for the entire period, preventing the continuous collection of urine. However, the increase in creatinine and BUN levels may indicate renal damage (Figure 5b and Supplementary Material S4) [38,40]. Liver stress was also reflected by the elevated levels of liver-derived metabolites, including total bilirubin and AST. Furthermore, histological examination of liver tissues revealed serositis, hepatitis, and necrosis in animals in the PAT group (Figure 3). These are consistent with liver impairments often seen in human sepsis [40,41]. Despite these findings, the porcine bilirubin levels associated with sepsis do not correlate with human values, instead these would be considered within the normal range [42]. The PAT group showed significant biochemical changes indicative of systemic inflammation and organ dysfunction. Post-injury, animals exhibited hyperlactatemia, reflecting impaired tissue oxygenation and metabolic stress. Glucose levels initially increased, followed by hypoglycemia, a pattern consistent with the hypermetabolic state and subsequent energy depletion seen in sepsis [11,38,43]. In addition, the immune response was characterized by increased levels of the proinflammatory cytokines IL-6, IL-10, IL-1β, and TNF-α [28]. This rapid increase in cytokine levels parallels that in humans during sepsis; elevated cytokine levels promote systemic inflammation and can lead to worsened outcomes such as organ failure and mortality [28,40].

### 4.3. Comparison to Other Animal Models

There are various animal models in which fecal peritonitis-induced sepsis is investigated. Notable differences are found in the induction of fecal peritonitis, the criteria applied to define sepsis, post-intervention treatment, and the follow-up period, which ranges from 8 h to 72 h [26,27]. 

Cecal ligation and puncture, a commonly used method for inducing fecal peritonitis, involves ligating the cecum to increase intraluminal pressure. The subsequent puncture of the organ results in fecal leakage of autologous intestinal bacteria into the peritoneal cavity. The size of the puncture determines the amount of soilage and, consequently, the severity of peritonitis and the development of sepsis [25,44,45,46]. Another method for peritonitis induction is the intraperitoneal implantation of bacterial fibrin clots, where specific bacteria are cultured externally and incorporated into fibrin clots. This method allows for precise determination of the type and exact quantity of bacteria [47,48]. Additionally, a commonly applied study design is the direct administration of fecal material through the direct injection of diluted feces, either autologous or allogenic, into the peritoneal cavity [27,29,49,50,51,52]. The quantity of fecal matter varies between studies, ranging from 0.5 g/kg [53] to 3 g/kg [54,55] with most studies using 1 g/kg [29,50,51,56,57]. In our study, we employed a novel approach that combines elements of the cecal ligation model and fecal matter application. This involved intestinal punctures simulating penetrating trauma from gunshots, with multiple intestinal injuries and discontinuity, presumably resulting in fecal matter leakage throughout the protocol, along with the additional application of diluted feces at 1 g/kg. Compared to other study designs, our model is the first to depict this specific scenario, offering a more realistic approach to peritoneal soilage after penetrating abdominal trauma.

Further, our model shows a high degree of effectiveness in replicating the key aspects of systemic inflammation and multi-organ dysfunction. The consistent patterns of hemodynamic changes, renal alterations and cytokine responses observed in various studies confirm the applicability of this porcine model in sepsis research. For example, O’Connell et al. developed a polytrauma peritonitis model that generates detectable bacteremia [23]. This model resulted in various abdominal injuries, as well as injuries to the bone, liver, spleen, and brain; expectedly, this caused major hemodynamic consequences. Similar to our study, there was an initial increase in HR, followed by a downward trend that never returned to the initial level [23]. In addition, more severe changes in pH and lactate levels, indicative of metabolic acidosis, were caused by the additional hemorrhagic component. O’Connell’s polytrauma-peritonitis model highlights the variability in physiologic responses depending on the severity and type of injury inflicted. This variability emphasizes the importance of selecting an appropriate animal model based on the specific research objectives [23]. In the study by Ji et al., the animals were observed in a state of septic shock, defined as MAP < 65 mmHg [28]. These animals required additional therapy with norepinephrine or vasopressin. Sepsis-induced renal dysfunction, characterized by increasing creatinine levels and decreased urine output, was demonstrated, particularly in the norepinephrine-treated group, further confirming the relevance of the current porcine model for the study of sepsis-related organ dysfunction. This comparison not only supports the validity of the porcine model but also provides a benchmark against which future models can be evaluated.

Additionally, our model demonstrated increased serum bilirubin, ALT, and AST levels, alongside systemic inflammation, as evidenced by elevated concentrations of TNF-α, IL-6, IL-10, and IL-1β. This immunological response is consistent with the general cytokine elevation reported in other studies and signifies early immune activation in septic shock, where TNF-α and IL-6 are key cytokines [15,26,29]. However, the variations in cytokine levels and peaks differ when compared to other fecal peritonitis studies.

Multiple studies have reported an increase in IL-6 levels beginning at 3 h after inoculation, with sustained rise until the end of their 12-h observation period [29,58,59]. This is in agreement with our findings, where the IL-6 level was significantly elevated until 32 h. By extending the observation period, we were able to demonstrate a reduction in the level of this cytokine, as part of is its physiological trajectory. Elevation of the IL-1β level was observed after 5 h by Park et al., with an increase until 12 h. In our study, we also noted elevated IL-1β levels that persisted until the study’s conclusion. TNF-α levels are heterogeneously described across studies. Park et al. reported an increase after 2 h, peaking at 3 h, while Laroye et al. noted a high level immediately following peritonitis induction, which decreased during the first 4 h [29,59]. Horak et al. found TNF-α levels to increase until the end of the 24-h observation period. In our study, the TNF-α level peaked at the 16-h timepoint and remained elevated until the end of our protocol. Differences in the role of TNF-α in animal models of sepsis are addressed in a systematic review by Kassassey et al., which highlights the unclear role of TNF-α and describes how TNF-α levels depend on multiple factors, such as sepsis induction [60].

The comparability of cytokine levels observed to those in other fecal peritonitis models is limited, as these models employed a different method of inducing peritonitis (direct application of fecal matter) and never encompassed an observation period of 72 h. Further, cytokine levels, in particular, are predominantly described in studies that induce sepsis by the injection of endotoxins. In these studies, the peak in cytokine level occurs closer to inoculation because foreign matter directly contacts immune cells in the bloodstream. Peaks in TNF-α and IL-10 levels are described 2 h after injection, and a peak in IL-6 level is described 4 h after injection [61,62]. This seems to contrast with our model; however, our blood draws were performed every 8 h so we could have missed these earlier peaks.

Further pathological findings included neutrophil accumulation and edema in the intestine, kidneys, and liver. By contrast, the fecal peritonitis model in the de Azevedo study, in which peritonitis was caused by the injection of 1.5 g/kg diluted feces into the peritoneal cavity, showed acute lung injury with a reduced P/F ratio (<300) [27]. This could not be reproduced in our model, as the animals never showed respiratory distress or a P/F ratio below 400. However, failure to replicate the acute lung injury and reduced P/F ratio observed suggests that our current porcine model may be less suitable for the study of respiratory complications associated with sepsis in the 72-h study period used. 

### 4.4. Diagnostic Scoring Systems

A multitude of studies have demonstrated the use of different of scoring systems in the diagnosis and evaluation of sepsis [63,64]. Our study further suggests a superiority of the NEWS2 score over SOFA score in assessing the condition of septic patients, further aligning with previously published studies [65]. 

Notwithstanding the persistent and substantial discrepancies between the two test groups (PAT group vs. Control group), the results of the SOFA and NEWS2 scores demonstrate temporal variations. While the septic state of the PAT group persisted at elevated levels, as indicated by the NEWS2 score, a clearer tendency toward normal values was recorded toward the end of the protocol using the SOFA score. This discrepancy may be attributable to the disparate parameters employed in the assessment of the scores. For instance, the SNORT score (category: Response to Offered Food and Treat) included in the SOFA score appears to have influenced the results in our study, as during the course of the protocol animals started to experience an escalation in appetite, resulting in a resumption of feeding behavior toward the end of the protocol, leading to higher scoring results. Furthermore, our protocol did not include removing the spleen. When interpreting the results, it is important to consider the role of the spleen in regenerating the immune and blood systems. This is reflected, in particular, in counts of blood platelets and white blood cells (WBCs) in the SOFA and SIRS scores (Figure 4d and Figure 5a).

Additionally, we did not develop the SOFA score ourselves; we merely modified it from the score established by Waterhouse and Fukuda et al. [34,35]. Score values generated independently based on our large cohort of baseline values may have been more accurate and better suited for evaluating the results of our study.

### 4.5. Limitations of This Study 

Swine have similar anatomy to humans but have known differences in the gut microbiome and gastrointestinal tract content [23,24,66,67]. These similarities provide a straightforward approach for inducing peritonitis and sepsis without prior bacterial growth or inoculation; however, the method of creating intraperitoneal sepsis poses difficulties in controlling its magnitude and making it more standardized [66]. Suggested guidelines for animal models in sepsis research recommend therapeutic intervention at the time of sepsis, e.g., fluid resuscitation and broad-spectrum antibiotics; however, with our goal of developing a model relevant to delayed evacuation on the battlefield, where these treatments may not be available, they are not utilized in our model [68]. Lastly, our study did not account for differences in sex; factors such as sex, age, and health may affect the severity of sepsis [39].

## 5. Conclusions

In our study, a reproducible and clinically relevant swine model of abdominal penetration trauma leading to fecal peritonitis-derived abdominal sepsis was successfully developed and validated. The model emphasizes the pathophysiologic cardiovascular, biochemical, and immunological changes associated with sepsis over a period of 72 h. The pathological condition was additionally validated using swine-specific adapted common diagnostic tools, such as SIRS criteria and SOFA and NEWS2 scores, as well as the expertise of different professionals. Our results underscore the importance of translational animal research that realistically mimics penetrating abdominal trauma, such as from gunshot wounds, as well as delayed patient evacuation and care. This creates pathophysiological changes that advance future possibilities for sepsis after abdominal trauma.

## Figures and Tables

**Table 1:** SIRS criteria for Yorkshire pigs: adapted SIRS criteria for Yorkshire pigs based on data from 48 healthy animals with matching CBC and DSI data from 3 protocols utilizing ± two standard deviations as the threshold for SIRS.

Parameter	Time of Day	Average	SD	SIRS (+2 SD)	SIRS (−2 SD)
HR [bpm]	Day	100	15.1	130	70
	Night	92	12.0	116	68
RR [bpm]	Day	22	3.9	29	14
	Night	16	3.1	23	10
Temp [°C]	Day	38.5	0.69	39.9	37.1
	Night	38.8	0.47	39.7	37.8
WBC [10^3^/µL]		19.1	3.5	26.2	12.1

**Table 2:** Sequential Organ Failure Assessment (SOFA) score: adapted SIRS criteria for Yorkshire pigs using values from Fukuda et al. 2019 [35] and Waterhouse et al. 2018 [34].

Sequential Organ Failure Assessment (SOFA) Score						
Organ System		Score (0–24)				
		0	1	2	3	4
Respiratory	PaO_2_/FiO_2_ ratio	≥400	<400	<300	<200	<100
Renal *	Creatine [mg/dL]	0.3–1.4	1.5–2.4	2.5–3.4	3.5–4.9	≥5.0
Hepatic *	Bilirubin [mg/dL]	0.3–0.6	0.7–2.0	2.1–5.0	5.1–10.0	>10.0
Hematological **	Platelet count [10^3^/µL]	>150	≤150	≤100	≤50	≤20
Cardiovascular *	MAP [mmHg]	>70	60–70	50–59	40–49	<40
Neurological **	SNORT	11–10	9–8	7–6	5–4	3–2
Sepsis = suspected or documented infection + SOFA score increase of ≥2 from baseline (indicates organ dysfunction)						

* Waterhouse et al., 2018; values from Yorkshire pigs [34]; ** Fukuda et al., 2019; values are from Sheep [35].

**Table 3:** Adapted values for National Early Warning Score 2 (NEWS2) [36] for Yorkshire pigs based on data from 48 healthy animals with matching DSI data from 3 protocols utilizing ± two standard deviations as the threshold for NEWS2.

Parameter	Time of Day	3	2	1	0	1	2	3
RR [bpm]	D	6		14	18–26		33	37
	N	4		10	13–20		26	29
SpO_2_ [%]		≤91	92–93	94–95	≥96	≤91		
Systolic BP [mmHg]	D	85	99	113	126–154			196
	N	83	95	108	120–145			183
HR [bpm]	D	40		70	85–115	130	145	161
	N	45		68	80–104	116	128	140
SNORT								<10
Temp [°C]	D	35.7		37.1	37.8–39.2	39.9	40.5	
	N	36.9		37.8	38.3–39.2	39.7	40.2	
A positive NEWS2 score is indicated by a sum of ≥5 or an extreme value variation, as shown by red highlighting [36].								

**Table 4:** Scoring system results for SIRS, SOFA, and NEWS2. Control (CON) group: *n* = 4. Penetrating Abdominal Trauma (PAT) group: *n* = 8.

Timepoint (Hours)	SIRS		SOFA		NEWS2	
	CON	PAT	CON	PAT	CON	PAT
0	0%	100%	50%	75%	25%	100%
8	0%	100%	25%	100%	0%	100%
16	0%	86%	0%	100%	0%	100%
24	0%	43%	0%	86%	0%	100%
32	0%	100%	0%	100%	0%	100%
40	0%	17%	0%	100%	0%	100%
48	0%	33%	0%	83%	0%	100%
56	0%	50%	0%	100%	0%	100%
64	0%	17%	0%	66%	0%	83%
72	0%	17%	0%	50%	0%	100%

## Data Availability

Data are provided in Supplementary Materials S3 and S4. Due to the size of the raw files, datasets are available upon request.

## Supplementary Materials

The following supporting information can be downloaded at: https://www.mdpi.com/article/10.3390/medicina61091523/s1, S1: Telemetric implants and Ponemah software analysis; S2: Swine Neurological Observed Response Test (SNORT); S3: Exact *p*-values for graphs; S4: Additional laboratory data table.

## Abbreviations

The following abbreviations are used in this manuscript:

AST	Aspartate Transaminase
BP	Blood Pressure
bpm	Breaths per Minute
BUN	Blood Urea Nitrogen
CT	Computed Tomography
DSI	Data Sciences International
HR	Heart Rate
IL	Interleukin
IM	Intramuscular
MAP	Mean Arterial Pressure
NEWS	National Early Warning Score
PAT	Penetrating Abdominal Trauma
SIRS	Systemic Inflammatory Response Syndrome
SNORT	Swine Neurological Observed Response Test
SOFA	Sequential Organ Failure Assessment
WBC	White Blood Cell

## References

[1] Gumeniuk K., Lurin I.A., Tsema I., Malynovska L., Gorobeiko M., Dinets A. (2023). Gunshot injury to the colon by expanding bullets in combat patients wounded in hybrid period of the Russian-Ukrainian war during 2014–2020. BMC Surg..

[2] Glasgow S.C., Steele S.R., Duncan J.E., Rasmussen T.E. (2012). Epidemiology of modern battlefield colorectal trauma: A review of 977 coalition casualties. J. Trauma Acute Care Surg..

[3] Watson J.D., Aden J.K., Engel J.E., Rasmussen T.E., Glasgow S.C. (2014). Risk factors for colostomy in military colorectal trauma: A review of 867 patients. Surgery.

[4] Cardi M., Ibrahim K., Alizai S.W., Mohammad H., Garatti M., Rainone A., Di Marzo F., La Torre G., Paschetto M., Carbonari L. (2019). Injury patterns and causes of death in 953 patients with penetrating abdominal war wounds in a civilian independent non-governmental organization hospital in Lashkargah, Afghanistan. World J. Emerg. Surg..

[5] Stockinger Z.T., Turner C.A., Gurney J.M. (2018). Abdominal trauma surgery during recent US combat operations from 2002 to 2016. J. Trauma Acute Care Surg..

[6] Turner C.A., Stockinger Z.T., Gurney J.M. (2017). Combat surgical workload in Operation Iraqi Freedom and Operation Enduring Freedom: The definitive analysis. J. Trauma Acute Care Surg..

[7] Rignault D.P. (1992). Abdominal trauma in war. World J. Surg..

[8] Seymour C.W., Gesten F., Prescott H.C., Friedrich M.E., Iwashyna T.J., Phillips G.S., Lemeshow S., Osborn T., Terry K.M., Levy M.M. (2017). Time to Treatment and Mortality during Mandated Emergency Care for Sepsis. N. Engl. J. Med..

[9] Coleman J.J., Zarzaur B.L. (2017). Surgical Management of Abdominal Trauma: Hollow Viscus Injury. Surg. Clin. N. Am..

[10] Riha G.M., Kiraly L.N., Diggs B.S., Cho S.D., Fabricant L.J., Flaherty S.F., Kuehn R., Underwood S.J., Schreiber M.A. (2013). Management of the open abdomen during the global war on terror. JAMA Surg..

[11] Jang J.H., Choi E., Kim T., Yeo H.J., Jeon D., Kim Y.S., Cho W.H. (2024). Navigating the Modern Landscape of Sepsis: Advances in Diagnosis and Treatment. Int. J. Mol. Sci..

[12] Maier S., Hauser H., Buhr H.J., Mischinger H.-J. (2016). Pathophysiologie des akuten Abdomens. Akutes Abdomen: Diagnose—Differenzialdiagnose—Erstversorgung—Therapie.

[13] Mullen P.G., Windsor A.C., Walsh C.J., Fowler A.A., Sugerman H.J. (1995). Tumor necrosis factor-alpha and interleukin-6 selectively regulate neutrophil function in vitro. J. Surg. Res..

[14] Damas P., Reuter A., Gysen P., Demonty J., Lamy M., Franchimont P. (1989). Tumor necrosis factor and interleukin-1 serum levels during severe sepsis in humans. Crit. Care Med..

[15] Chousterman B.G., Swirski F.K., Weber G.F. (2017). Cytokine storm and sepsis disease pathogenesis. Semin. Immunopathol..

[16] Bone R.C., Balk R.A., Cerra F.B., Dellinger R.P., Fein A.M., Knaus W.A., Schein R.M., Sibbald W.J. (1992). Definitions for sepsis and organ failure and guidelines for the use of innovative therapies in sepsis. The ACCP/SCCM Consensus Conference Committee. American College of Chest Physicians/Society of Critical Care Medicine. Chest.

[17] Kaukonen K.M., Bailey M., Pilcher D., Cooper D.J., Bellomo R. (2015). Systemic inflammatory response syndrome criteria in defining severe sepsis. N. Engl. J. Med..

[18] Vincent J.L., Sakr Y., Sprung C.L., Ranieri V.M., Reinhart K., Gerlach H., Moreno R., Carlet J., Le Gall J.R., Payen D. (2006). Sepsis in European intensive care units: Results of the SOAP study. Crit. Care Med..

[19] Singer M., Deutschman C.S., Seymour C.W., Shankar-Hari M., Annane D., Bauer M., Bellomo R., Bernard G.R., Chiche J.D., Coopersmith C.M. (2016). The Third International Consensus Definitions for Sepsis and Septic Shock (Sepsis-3). JAMA.

[20] Lambden S., Laterre P.F., Levy M.M., Francois B. (2019). The SOFA score-development, utility and challenges of accurate assessment in clinical trials. Crit. Care.

[21] Royal College of Physicians (2017). National Early Warning Score (NEWS) 2: Standardising the Assessment of Acute-Illness Severity in the NHS.

[22] Qiu X., Yu-Peng L., Zhou R.-X. (2023). SIRS, SOFA, qSOFA, and NEWS in the diagnosis of sepsis and prediction of adverse outcomes: A systematic review and meta-analysis. Expert Rev. Anti-Infect. Ther..

[23] O’Connell R.L., Wakam G.K., Siddiqui A., Williams A.M., Graham N., Kemp M.T., Chtraklin K., Bhatti U.F., Shamshad A., Li Y. (2021). Development of a large animal model of lethal polytrauma and intra-abdominal sepsis with bacteremia. Trauma Surg. Acute Care Open.

[24] Hildebrand F., Andruszkow H., Huber-Lang M., Pape H.-C., van Griensven M. (2013). Combined Hemorrhage/Trauma Models in Pigs—Current State and Future Perspectives. Shock.

[25] Dejager L., Pinheiro I., Dejonckheere E., Libert C. (2011). Cecal ligation and puncture: The gold standard model for polymicrobial sepsis?. Trends Microbiol..

[26] Zurek-Leffers F.M., Lehmann F., Brabenec L., Kintrup S., Hellenthal K.E.M., Mersjann K., Kneifel F., Hessler M., Arnemann P.H., Kampmeier T.G. (2023). A model of porcine polymicrobial septic shock. Intensive Care Med. Exp..

[27] de Azevedo L.C., Park M., Noritomi D.T., Maciel A.T., Brunialti M.K., Salomão R. (2007). Characterization of an animal model of severe sepsis associated with respiratory dysfunction. Clinics.

[28] Ji M.H., Yang J.J., Wu J., Li R.Q., Li G.M., Fan Y.X., Li W.Y. (2012). Experimental sepsis in pigs--effects of vasopressin on renal, hepatic, and intestinal dysfunction. Ups. J. Med. Sci..

[29] Park I., Lee J.H., Jang D.H., Kim D., Chang H., Kwon H., Kim S., Kim T.S., Jo Y.H. (2019). Characterization of Fecal Peritonitis-Induced Sepsis in a Porcine Model. J. Surg. Res..

[30] Barker S.J., Gamel D.M., Tremper K.K. (1987). Cardiovascular effects of anesthesia and operation. Crit. Care Clin..

[31] Cicero L., Fazzotta S., Palumbo V.D., Cassata G., Lo Monte A.I. (2018). Anesthesia protocols in laboratory animals used for scientific purposes. Acta Biomed..

[32] Pluschke A.M., Simmons G.S., Keates H.L., Cameron R.D.A., Zhang D., Wright J.D., Williams B.A. (2017). An updated method for the jugular catheterization of grower pigs for repeated blood sampling following an oral glucose tolerance test. Lab. Anim..

[33] Burmeister D.M., McIntyre M.K., Baker B.A., Rizzo J.A., Brown A., Natesan S., Chung K.K., Christy R.J. (2016). Impact of Isolated Burns on Major Organs: A Large Animal Model Characterized. Shock.

[34] Waterhouse A., Leslie D.C., Bolgen D.E., Lightbown S.L., Dimitrakakis N., Cartwright M.J., Seiler B.T., Lightbown K.R., Smith K.P., Lombardo P. (2018). Modified Clinical Monitoring Assessment Criteria for Multi-Organ Failure during Bacteremia and Sepsis Progression in a Pig Model. Advan Crit. Care Med..

[35] Fukuda S., Ihara K., Bohannon J.K., Hernandez A., Patil N.K., Luan L., Stothers C., Stark R., Prough D.S., Herndon D.N. (2020). Monophosphoryl Lipid a Attenuates Multiorgan Dysfunction During Post-Burn Pseudomonas Aeruginosa Pneumonia in Sheep. Shock.

[36] Prytherch D.R., Smith G.B., Schmidt P.E., Featherstone P.I. (2010). ViEWS—Towards a national early warning score for detecting adult inpatient deterioration. Resuscitation.

[37] Gaeth C., Duarte J., Rodriguez A., Powers A., Stone R. (2024). Observational Analysis of Point-of-Care Lactate Plus™ Meter in Preclinical Trauma Models. Diagnostics.

[38] Seymour C.W., Liu V.X., Iwashyna T.J., Brunkhorst F.M., Rea T.D., Scherag A., Rubenfeld G., Kahn J.M., Shankar-Hari M., Singer M. (2016). Assessment of Clinical Criteria for Sepsis: For the Third International Consensus Definitions for Sepsis and Septic Shock (Sepsis-3). JAMA.

[39] Hayase N., Doi K. (2024). How Do We Bridge the Gap Between Animal Models of Sepsis and Patients?. Kidney360.

[40] Angus D.C., van der Poll T. (2013). Severe sepsis and septic shock. N. Engl. J. Med..

[41] Woźnica E.A., Inglot M., Woźnica R.K., Łysenko L. (2018). Liver dysfunction in sepsis. Adv. Clin. Exp. Med..

[42] Rawal R., Kharangarh P.R., Dawra S., Tomar M., Gupta V., Pundir C.S. (2020). A comprehensive review of bilirubin determination methods with special emphasis on biosensors. Process Biochem..

[43] Van Cromphaut J.S., Vanhorebeek I., den Berghe G.V. (2008). Glucose Metabolism and Insulin Resistance in Sepsis. Curr. Pharm. Des..

[44] Kato T., Hussein M.H., Sugiura T., Suzuki S., Fukuda S., Tanaka T., Kato I., Togari H. (2004). Development and characterization of a novel porcine model of neonatal sepsis. Shock.

[45] Rittirsch D., Huber-Lang M.S., Flierl M.A., Ward P.A. (2009). Immunodesign of experimental sepsis by cecal ligation and puncture. Nat. Protoc..

[46] Kieslichova E., Rocen M., Merta D., Kudla M., Splichal I., Cap J., Viklicky O., Gürlich R. (2013). The effect of immunosuppression on manifestations of sepsis in an animal model of cecal ligation and puncture. Transplant. Proc..

[47] Goldfarb R.D., Marton A., Szabó É., Virág L., Salzman A.L., Glock D., Akhter I., McCarthy R., Parrillo J.E., Szabó C. (2002). Protective effect of a novel, potent inhibitor of poly(adenosine 5′-diphosphate-ribose) synthetase in a porcine model of severe bacterial sepsis. Crit. Care Med..

[48] Goldfarb R.D., Parker T.S., Levine D.M., Glock D., Akhter I., Alkhudari A., McCarthy R.J., David E.M., Gordon B.R., Saal S.D. (2003). Protein-free phospholipid emulsion treatment improved cardiopulmonary function and survival in porcine sepsis. Am. J. Physiol. Regul. Integr. Comp. Physiol..

[49] Hyun H., Lee M.S., Park I., Ko H.S., Yun S., Jang D.H., Kim S., Kim H., Kang J.H., Lee J.H. (2021). Analysis of Porcine Model of Fecal-Induced Peritonitis Reveals the Tropism of Blood Microbiome. Front. Cell Infect. Microbiol..

[50] Al-Obeidallah M., Jarkovská D., Valešová L., Horák J., Jedlička J., Nalos L., Chvojka J., Švíglerová J., Kuncová J., Beneš J. (2021). SOFA Score, Hemodynamics and Body Temperature Allow Early Discrimination between Porcine Peritonitis-Induced Sepsis and Peritonitis-Induced Septic Shock. J. Pers. Med..

[51] Jarkovska D., Markova M., Horak J., Nalos L., Benes J., Al-Obeidallah M., Tuma Z., Sviglerova J., Kuncova J., Matejovic M. (2018). Cellular Mechanisms of Myocardial Depression in Porcine Septic Shock. Front. Physiol..

[52] Ljungdahl M., Rasmussen I., Haglund U. (1999). Intestinal blood flow and intramucosal pH in experimental peritonitis. Shock.

[53] Hauser B., Barth E., Bassi G., Simon F., Gröger M., Öter S., Speit G., Ploner F., Möller P., Wachter U. (2009). Hemodynamic, metabolic, and organ function effects of pure oxygen ventilation during established fecal peritonitis-induced septic shock. Crit. Care Med..

[54] Garcia B., Su F., Dewachter L., Favory R., Khaldi A., Moiroux-Sahraoui A., Annoni F., Vasques-Nóvoa F., Rocha-Oliveira E., Roncon-Albuquerque R. (2022). Myocardial effects of angiotensin II compared to norepinephrine in an animal model of septic shock. Crit. Care.

[55] Ferrario M., Brunelli L., Su F., Herpain A., Pastorelli R. (2019). The Systemic Alterations of Lipids, Alanine-Glucose Cycle and Inter-Organ Amino Acid Metabolism in Swine Model Confirms the Role of Liver in Early Phase of Septic Shock. Front. Physiol..

[56] Marx G., Cobas Meyer M., Schuerholz T., Vangerow B., Gratz K.F., Hecker H., Sümpelmann R., Rueckoldt H., Leuwer M. (2002). Hydroxyethyl starch and modified fluid gelatin maintain plasma volume in a porcine model of septic shock with capillary leakage. Intensive Care Med..

[57] Simon F., Giudici R., Scheuerle A., Gröger M., Asfar P., Vogt J.A., Wachter U., Ploner F., Georgieff M., Möller P. (2009). Comparison of cardiac, hepatic, and renal effects of arginine vasopressin and noradrenaline during porcine fecal peritonitis: A randomized controlled trial. Crit. Care.

[58] Laroye C., Lemarié J., Boufenzer A., Labroca P., Cunat L., Alauzet C., Groubatch F., Cailac C., Jolly L., Bensoussan D. (2018). Clinical-grade mesenchymal stem cells derived from umbilical cord improve septic shock in pigs. Intensive Care Med. Exp..

[59] Horak J., Nalos L., Martinkova V., Tegl V., Vistejnova L., Kuncova J., Kohoutova M., Jarkovska D., Dolejsova M., Benes J. (2020). Evaluation of Mesenchymal Stem Cell Therapy for Sepsis: A Randomized Controlled Porcine Study. Front. Immunol..

[60] Kassasseya C., Torsin L.I., Musset C., Benhamou M., Chaudry I.H., Cavaillon J.M., Grall N., Monteiro R., de Chaisemartin L., Longrois D. (2024). Divergent effects of tumor necrosis factor (TNF) in sepsis: A meta-analysis of experimental studies. Crit. Care.

[61] Wilske F., Skorup P., Hanslin K., Janols H., Larsson A., Lipcsey M., Sjölin J. (2023). Enhanced bacterial clearance in early secondary sepsis in a porcine intensive care model. Sci. Rep..

[62] Ebdrup L., Krog J., Granfeldt A., Larsen P., Vestergaard C., Hokland M., Tønnesen E. (2008). Leukocyte, plasma, and organ-associated cytokine profiles in an animal model of acute inflammation. APMIS.

[63] Castello L.M., Gavelli F. (2024). Sepsis scoring systems: Mindful use in clinical practice. Eur. J. Intern. Med..

[64] Oduncu A.F., Kıyan G.S., Yalçınlı S. (2021). Comparison of qSOFA, SIRS, and NEWS scoring systems for diagnosis, mortality, and morbidity of sepsis in emergency department. Am. J. Emerg. Med..

[65] Usman O.A., Usman A.A., Ward M.A. (2019). Comparison of SIRS, qSOFA, and NEWS for the early identification of sepsis in the Emergency Department. Am. J. Emerg. Med..

[66] Poli-de-Figueiredo L.F., Garrido A.G., Nakagawa N., Sannomiya P. (2008). Experimental models of sepsis and their clinical relevance. Shock.

[67] Chalupova M., Horak J., Kramna L., Nalos L., Stengl M., Chudejova K., Kraftova L., Cinek O., Klein P., Matejovic M. (2022). Gut microbiome diversity of porcine peritonitis model of sepsis. Sci. Rep..

[68] Osuchowski M.F., Ayala A., Bahrami S., Bauer M., Boros M., Cavaillon J.M., Chaudry I.H., Coopersmith C.M., Deutschman C.S., Drechsler S. (2018). Minimum Quality Threshold in Pre-Clinical Sepsis Studies (MQTiPSS): An International Expert Consensus Initiative for Improvement of Animal Modeling in Sepsis. Shock.

