# Supplementary material for: Modeling Sepsis: Establishment and Validation of a 72-Hour Swine Model of Penetrating Abdominal Trauma
Source: Medicina (Kaunas). 2025 Aug 25;61(9):1523. doi: 10.3390/medicina61091523 (PMC12471421; doi:10.3390/medicina61091523)
Supplement: Supplementary file 1 [file medicina-61-01523-s001.zip › Supplemental material S1.pdf]

Supplementary Material S1 Telemetric Implants and Ponemah Software Analysis

Summary: Detailed information pertaining to the collection of data from the telemetric devices and the settings used in the analysis software.

PhysioTel M11 telemetric implants (Data Sciences International, DSI) routinely collected blood pressure at 500 Hz and temperature at 1 Hz. Signal strength between the transceiver and implant was also recorded (1 Hz). Ambient pressure was recorded at 1 Hz using a remote barometer module (used to calculate barometric offset). Computed respiration rate (bpr, blood pressure, respiration) was calculated at 50 Hz. All data was exported in Ponemah v. 6.51 (DSI) following a data reduction step to consolidate the data to 1 data point per hour (1/3600 Hz).

Data processing and analysis was conducted using Ponemah v. 6.51. Since Ponemah does not have default settings for *Sus scrofa*, we optimized the settings based on physiologically, statistically, or mathematically relevant numbers. To aid in confirming the accuracy of our calculations, we compared the values derived in Ponemah to the recorded values during procedures in the medical anesthesia report. We utilized the Activity (Act.), Ambient Pressure (APR), Blood Pressure (Pressure), Signal Strength (SS%), and Temperature (Temp) channels, as well as the derived channel, Respiration (Bpr). Bad data marks were not merged across channels and unless stated, the defaults were used for all other settings.

The changes to the analysis settings in Ponemah (including Standard Attributes, Advanced Attributes, Offsets, and Noise) used for our data are indicated below:

<u>Pressure:</u> Minimum Pulse Height = 5 (mmHg); Systolic Validation Time = 100 (ms); Non Detection Time = 50 (ms); Percent Recovery = 70%. Low Pass Filter = 10.0 (Hz). Minimum Signal Value = 15 (mmHg), Maximum Signal Value = 300 (mmHg), Minimum Heart Rate = 50 (bpm), Maximum Heart Rate = 300 (bpm), Minimum Good Data Time = 5 (s).

<u>Respiration (Bpr):</u> Minimum Pulse Height = 2 (mmHg); Pressure Drop = 1 (mmHg); Respiration Smoothing = 750 (s). Minimum Breath Rate = 8 (bpm), Maximum Breath Rate = 75 (bpm), Minimum Good Data Time = 0 (s).

<u>Temperature:</u> Minimum Signal Value = 20 (°C); Maximum Signal Value = 45 (°C); Minimum Good Data Time = 0 (s).

<u>Activity:</u> Minimum Signal Value = 0 (counts); Maximum Signal Value = 1000 (counts); Minimum Good Data Time = 0 (s).

<u>Signal Strength:</u> Minimum Signal Value = 0 (%), Maximum Signal Value = 100 (%), Minimum Good Data Time = 0 (s).

<u>APR</u>: All default settings.
