# Supplementary material for: Modeling Sepsis: Establishment and Validation of a 72-Hour Swine Model of Penetrating Abdominal Trauma
Source: Medicina (Kaunas). 2025 Aug 25;61(9):1523. doi: 10.3390/medicina61091523 (PMC12471421; doi:10.3390/medicina61091523)
Supplement: Supplementary file 1 [file medicina-61-01523-s001.zip › Supplemental material S2.pdf]

**Supplementary Material S2** Swine Neurological Observed Response Test (SNORT)

Summary: Swine Neurological Observed Response Test for the assessment of neurological status in Swine.

| Category | Score | Description |
|---|---|---|
| **Response to Approach** | 4 | Responds to approach (stands up) |
| | 2 | Eye follow, head movement, no standing |
| | 1 | No response |
| **Natural Behavior** | 4 | Respond to normal human voice |
| | 2 | Respond to clapping hands |
| | 1 | No response |
| **Response to Offered Food Treat** | 3 | Takes treat and eats immediately |
| | 2 | Takes treat but drops it with no interest |
| | 1 | Smells treat but doesn't try to take it |
| | 0 | No response |
| **Total Score** | 2-11 | |
